# Mesenchymal stem cells against intestinal ischemia–reperfusion injury: a systematic review and meta-analysis of preclinical studies

**DOI:** 10.1186/s13287-022-02896-y

**Published:** 2022-05-26

**Authors:** Yajing Shi, Xiaolan Zhang, Zhanhai Wan, Xin Liu, Feng Chen, Jianmin Zhang, Yufang Leng

**Affiliations:** 1 The First Clinical Medical College, Lanzhou University, No. 199, Donggang Road West, Chengguan District, Lanzhou, Gansu China; 2 The Department of Anesthesiology, Gansu Provincial Maternity and Child-Care Hospital, No. 143, Qilihe North Street, Qilihe District, Lanzhou, Gansu China; 3 The Department of Anesthesiology, The First Hospital of Lanzhou University, No. 1, Donggang Road West, Chengguan District, Lanzhou, Gansu China

**Keywords:** Intestinal ischemia–reperfusion injury, Mesenchymal stem cells, Systematic Review and meta-analysis, Preclinical studies

## Abstract

**Background:**

Intestinal ischemia–reperfusion injury (IRI) causes localized and distant tissue lesions. Multiple organ failure is a common complication of severe intestinal IRI, leading to its high rates of morbidity and mortality. Thus far, this is poorly treated, and there is an urgent need for new more efficacious treatments. This study evaluated the beneficial effects of mesenchymal stem cells (MSCs) therapy on intestinal IRI using many animal experiments.

**Methods:**

We conducted a comprehensive literature search from 4 databases: Pubmed, Embase, Cochrane library, and Web of science. Primary outcomes included the survival rate, Chiu’s score, intestinal levels of IL-6, TNF-α and MDA, as well as serum levels of DAO, D-Lactate, and TNF-α. Statistical analysis was carried out using Review Manager 5.3.

**Results:**

It included Eighteen eligible researches in the final analysis. We demonstrated that survival rates in animals following intestinal IRI were higher with MSCs treatment compared to vehicle treatment. Besides, MSCs treatment attenuated intestinal injury caused by IRI, characterized by lower Chiu’s score (− 1.96, 95% CI − 2.72 to − 1.19, *P* < 0.00001), less intestinal inflammation (IL-6 (− 2.73, 95% CI − 4.19 to − 1.27, *P* = 0.0002), TNF-α (− 3.00, 95% CI − 4.74 to − 1.26, *P* = 0.0007)) and oxidative stress (MDA (− 2.18, 95% CI − 3.17 to − 1.19, *P* < 0.0001)), and decreased serum levels of DAO (− 1.39, 95% CI − 2.07 to − 0.72, *P* < 0.0001), D-Lactate (− 1.54, 95% CI − 2.18 to − 0.90, *P* < 0.00001) and TNF-α (− 2.42, 95% CI − 3.45 to − 1.40, *P* < 0.00001). The possible mechanism for MSCs to treat intestinal IRI might be through reducing inflammation, alleviating oxidative stress, as well as inhibiting the apoptosis and pyroptosis of the intestinal epithelial cells.

**Conclusions:**

Taken together, these studies revealed that MSCs as a promising new treatment for intestinal IRI, and the mechanism of which may be associated with inflammation, oxidative stress, apoptosis, and pyroptosis. However, further studies will be required to confirm these findings.

**Supplementary Information:**

The online version contains supplementary material available at 10.1186/s13287-022-02896-y.

## Introduction

Ischemia–reperfusion injury (IRI) is a common clinical problem in which ischemic injury of a tissue or organ is exacerbated by restoring blood flow. IRI occurs in various organs and tissues, such as the liver, kidney, brain, heart, lung, retina, and intestine. Intestinal IRI is a major complication of severe trauma, burns, infection, shock, and cardiopulmonary insufficiency [1]. Intestinal IRI damages intestinal epithelial cells (IECs) and causes intestinal barrier dysfunction, allowing bacterial translocation [2]. Further, severe intestinal IRI can disrupt the normal architecture and function of multiple organs, which eventually results in endotoxemia, systemic inflammatory response syndrome (SIRS), and even multiple organ dysfunction and failure. Thus, intestinal IRI contributes to unacceptably high morbidity and mortality rates in clinical settings [3]. Until now, there is no ideal treatment for it [4], and the development of novel agents for it remains a critical challenge.

Mesenchymal stem cells (MSCs) are derived from the mesoderm. They exist in a variety of organs and tissues, including bone marrow, umbilical cord, placenta, and adipose tissue. MSCs are pluripotent cells with extensive self-renewal potential and can differentiate into various non-hematopoietic cells (osteoblast, chondrocyte, myocyte, adipocyte, hepatocyte, fibroblasts, enterocyte, neurocyte, endothelium, tendon, and ligament) under certain conditions [5]. In recent years, a growing body of research has reported MSCs can contribute to healing of injured tissues and curing many diseases [6–10] by inhibition of apoptosis, inflammation, and fibrosis, promotion of angiogenesis, release of repair factors, and immunomodulation [11, 12].

In injured intestine tissue, intestinal stem cells differentiate into IECs to replace the dying or damaged ones, which restore intestinal barrier function [13]. It seems likely that MSCs could be a potential approach to promote intestinal barrier function during intestinal IRI. Despite a vast literature on the relationship between MSCs and intestine IRI in animals, they used different experimental designs and showed contradictory results. Thus, this research evaluated the effectiveness of MSCs in animals following intestinal IRI.

## Methods

### Search strategy

We carried out a systematic review and meta-analysis in accordance with the Preferred Reporting Items for Systematic Reviews and Meta-Analyses (PRISMA) guidelines [14]. We used “mesenchymal stem cell,” “intestinal ischemia–reperfusion,” and “nonhuman” as keywords to search PubMed, Embase, Cochrane Library, and Web of Science databases (from inception to August 25th, 2021) (see Additional file 1). And this search was finished by two authors (YJ Shi and ZH Wan) independently.

### Inclusion and exclusion criteria

Inclusion criteria were as follows: (1) nonhuman studies; (2) animal models of intestinal IRI; (3) aim to investigate the efficacy of MSCs in intestinal IRI. Exclusion criteria were as follows: (1) duplicate publication; (2) animal models of intestinal IRI were not induced by superior mesenteric artery (SMA) occlusion and deocclusion [15]; (3) study the protective effect of MSCs on other organs than the small intestine; (4) no available data.

### Study selection and data extraction

Two authors (YJ Shi and XL Zhang) screened titles and abstracts based on the above inclusion and exclusion criteria. Eventually, eighteen studies were eligible for this meta-analysis. X Liu and F Chen independently read the included articles to extract the experimental details and data as follows: (1) study’s characteristics (i.e., first author’s name, country, publication year); (2) animals (i.e., species, gender, age, weight); (3) intestinal IRI (i.e., duration of SMA occlusion and deocclusion) (4) MSCs (i.e., type, dosage, administration route and timing); (5) anesthetics.

### Assessment of study quality

Two authors (YJ Shi and JM Zhang) independently assessed the quality of included studies using the Cochrane risk of bias tool [16].

### Statistical analysis

We used Review Manager 5.3 to conduct this meta-analysis. Continuous variables were expressed as the mean ± standard deviation (SD), and dichotomous variables (survival rate) were expressed as risk ratios. We converted medians and interquartile ranges to means and standard deviations according to the formula [17] for subsequent analyses. Statistical heterogeneity was assessed using the chi-squared (χ^2^) statistical test (the α-level for statistical significance was 0.05) and the inconsistency index (Ι^2^) statistic. Due to anticipated heterogeneity, meta-analysis was performed using a random-effects model. In addition, we performed subgroup analysis in order to better understand the outcomes of this study. For all analyses, *P* < 0.05 was considered statistically significant.

## Results

### Study screen

**Fig. 1:**
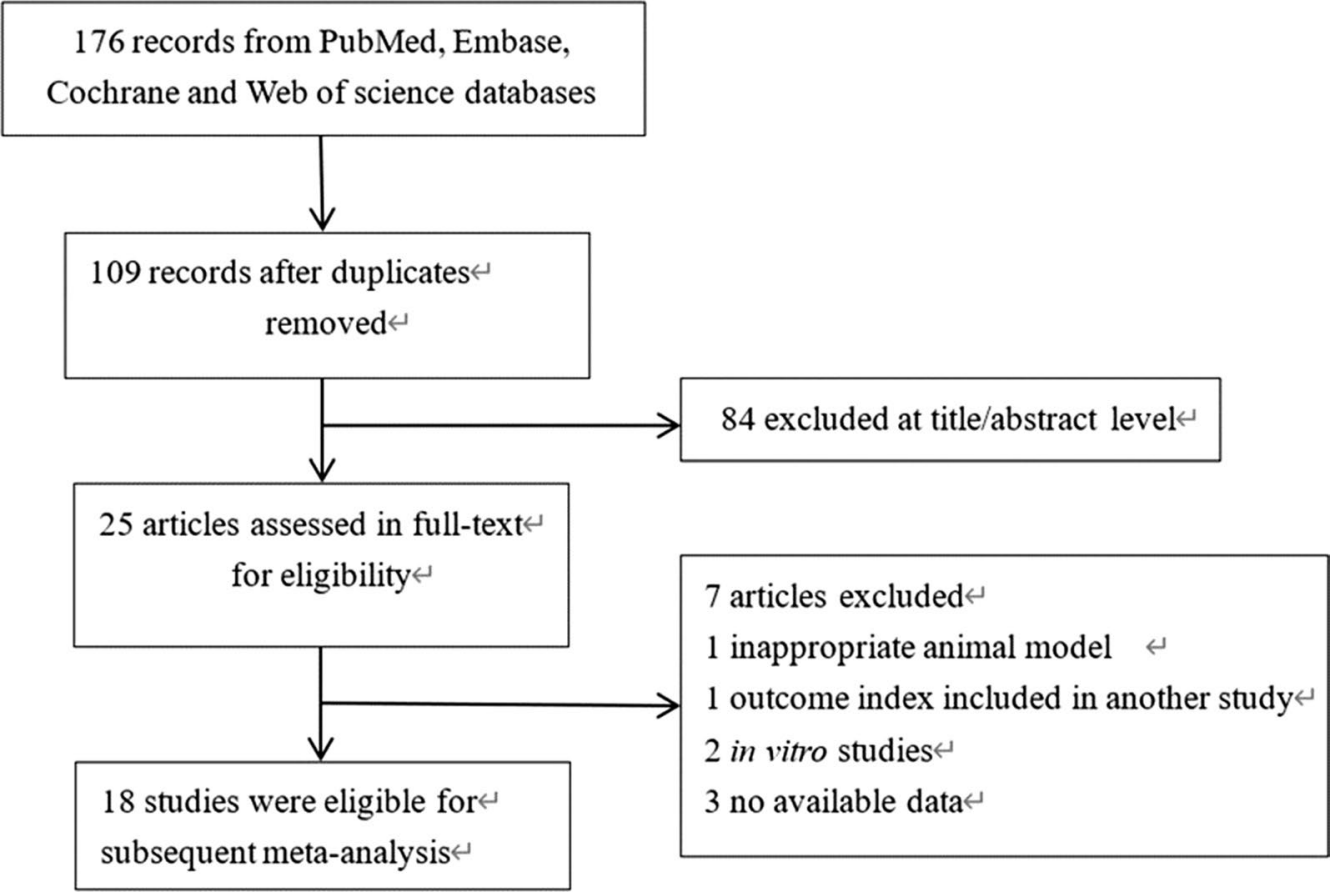
Flowchart of the article screening process

The search strategy retrieved 176 articles from the four databases, of which 109 were excluded as duplicates. After title and abstract screening, 25 articles were identified and underwent review of the full text. Ultimately, 7 studies were included in the meta-analysis [4, 18–34] (Fig. 1).

### Characteristics and quality of included studies

**Fig. 2:**
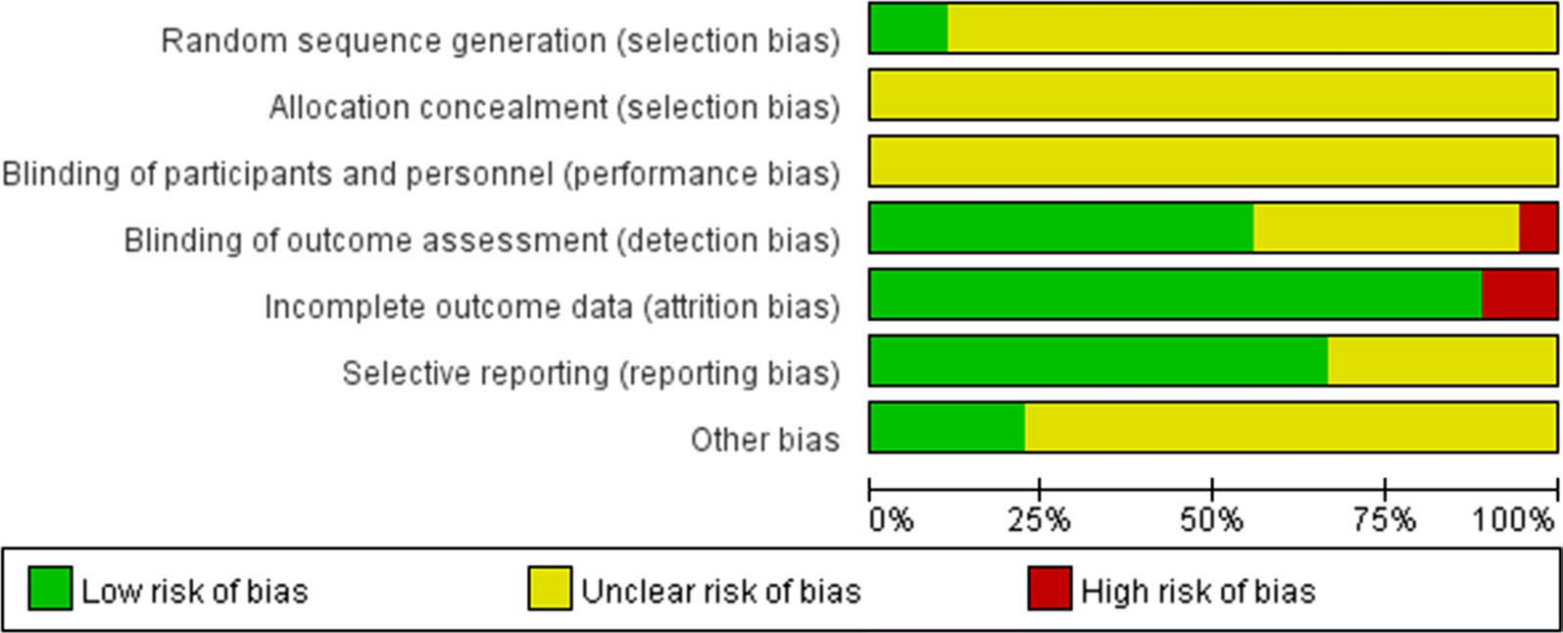
Overall quality of the included studies assessed by Cochrane risk of bias assessment tool

**Fig. 3:**
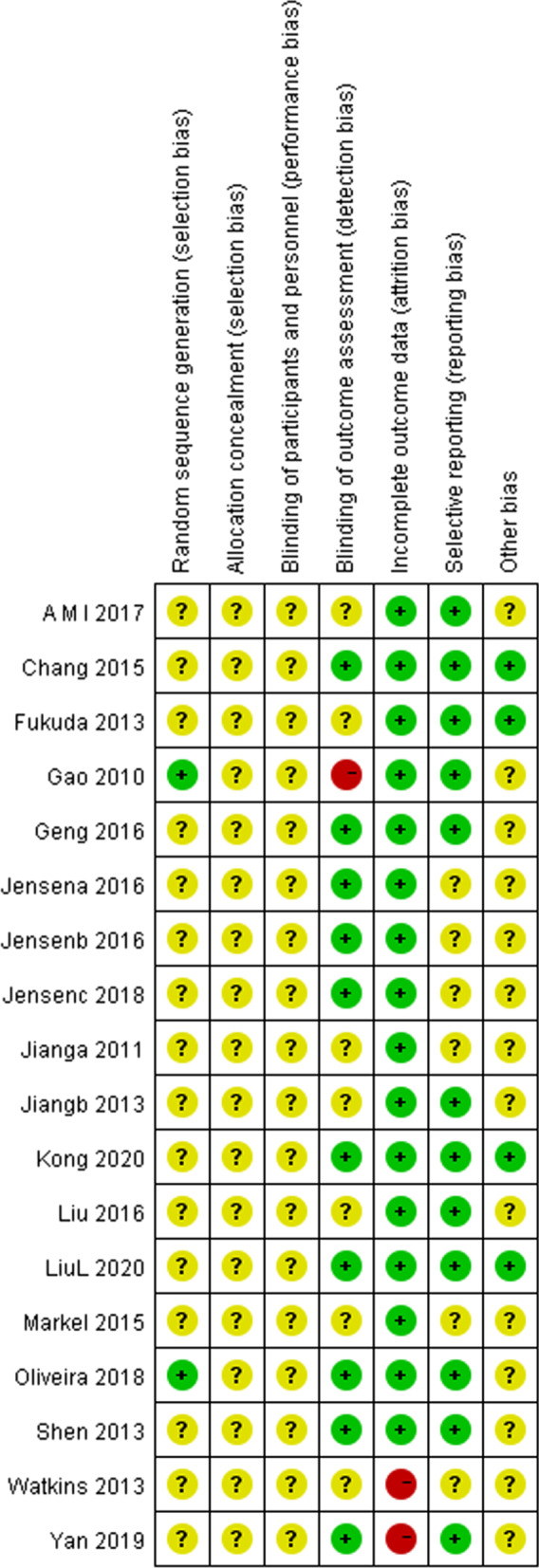
Risk of bias within studies assessed by Cochrane risk of bias assessment tool

The detailed characteristics of the included studies are provided in Table 1. The major limitation was that most of those did not clearly report random sequence generation, allocation concealment, and blinding of participants and personnel (Figs. 2 and 3).

**Table 1:** Characteristics of included studies

Author	Year	Country	Species/gender	Age/weight	I/R duration	Anesthetic	MSCs type /dosage	Administration	Timing of MSCs (post-reperfusion)
AMI	2017	Turkey	SD rats/f	200–250 g	45 min/1, 4, 7 d	Xylazine/ketamine	BM, allogeneic, 0.5/1 × 10^6^	Inferior vena cava/local injection	Immediately
Chang	2015	Taiwan	SD rats/m	325–350 g	30 min/3 d	Unclear	AD, autologous, 1.2 × 10^6^	Intravenous and local injection	Immediately
Fukuda	2013	Japan	ICR mice	Unclear	60 min/2, 6 h	Unclear	AD, autologous, 1 × 10^5^/10^6^	i.p	Immediately
Gao	2010	China	Wistar rats/m	≈ 200 g	20 min/0.5, 1, 3, 7, 14, 28 d	Unclear	BM, allogeneic, 1 × 10^6^	Caudal vein	Immediately
Geng	2016	China	SD rats/m	180–220 g	30 min/2 h	Unclear	BM, allogeneic, 1 × 10^7^	Caudal vein	Immediately
Jensena	2016	USA	C57Bl6 mice/m	8–12 w, 20–30 g	60 min/12, 24 h	Isoflurane	hAD, 2 × 10^6^	i.p	Unclear
Jensenb	2016	USA	C57Bl6 mice/m	8–12 w, 20–30 g	60 min/12, 24 h	Isoflurane	hAD, hUD, 2 × 10^6^	i.p	Unclear
Jensen	2018	USA	C57Bl6 mice/m	8–12 w	60 min/24 h	Isoflurane	hUD, 2 × 10^6^	i.p	Immediately
Jiang	2011	China	SD rats/f	180–200 g	45 min/4, 7 d	Ketamine	BM, allogeneic (m), 1 × 10^7^	Local injection	Immediately
Jiang	2013	China	SD rats/f	180–220 g	45 min/1, 4, 7 d	Ketamine	BM, allogeneic (m), 1 × 10^7^	Local injection	Immediately
Kong	2020	China	SD rats/m	250–300 g	30 min/72 h	Pentobarbital	AD, allogeneic, 2 × 10^6^	Caudal vein	Unclear
Liu	2016	China	SD rats	6–8 w/180–210 g	30 min/2, 6, 24, 72, 120 h	Unclear	BM, allogeneic, 5 × 10^6^	Local injection	Unclear
Liu	2020	China	C57Bl6 mice	20–25 g	60 min/2 d	Pentobarbital	AD, allogeneic, 5 × 10^6^	Local injection	Unclear
Markel	2015	USA	C57Bl6 mice/m	8–12 w/20-30 g	60 min/6 h	Isoflurane	hBM, 2 × 10^4^/10^5^/10^6^	i.p	Unclear
Oliveira	2018	Brazil	NZ rabbits	≈ 10 w/≈ 3 kg	2 h/3, 7 d	Xylazine, ketamine, tramadol, isoflurane	AD, allogeneic, 1.2 × 10^6^	Marginal auricular vein	5 h
Shen	2013	China	SD rats/m	120–200 g	30 min/2, 6, 24, 72, 144 h	Chloral hydrate	BM, allogeneic, 1 × 10^7^	Local injection	Immediately
Watkins	2013	USA	FVB mice/m	8–10 w/≈ 20 g	60 min/24 h	Isoflurane	BM/AF (from C57Bl6 mice), 1 × 10^6^	i.p	2 h
Yan	2019	China	SD rats/m	unclear	60 min/1, 7 d	Pentobarbital	BM, allogeneic, 1 × 10^7^	i.p	Unclear

SD: Sprague–Dawley; f: female; m: male; MSCs: mesenchymal stem cells; BM: Bone marrow; hBM: Human BM; AD: adipose-derived; AF: amniotic fluid; hUD: Human umbilical Cord; NZ: New Zealand; i.p.: intraperitoneal; min: minute(s); h: hour(s); d: day(s); w: week(s); g: gram(s); kg: kilogram(s); USA: United States of America

### Effectiveness

#### Survival rate

**Fig. 4:**
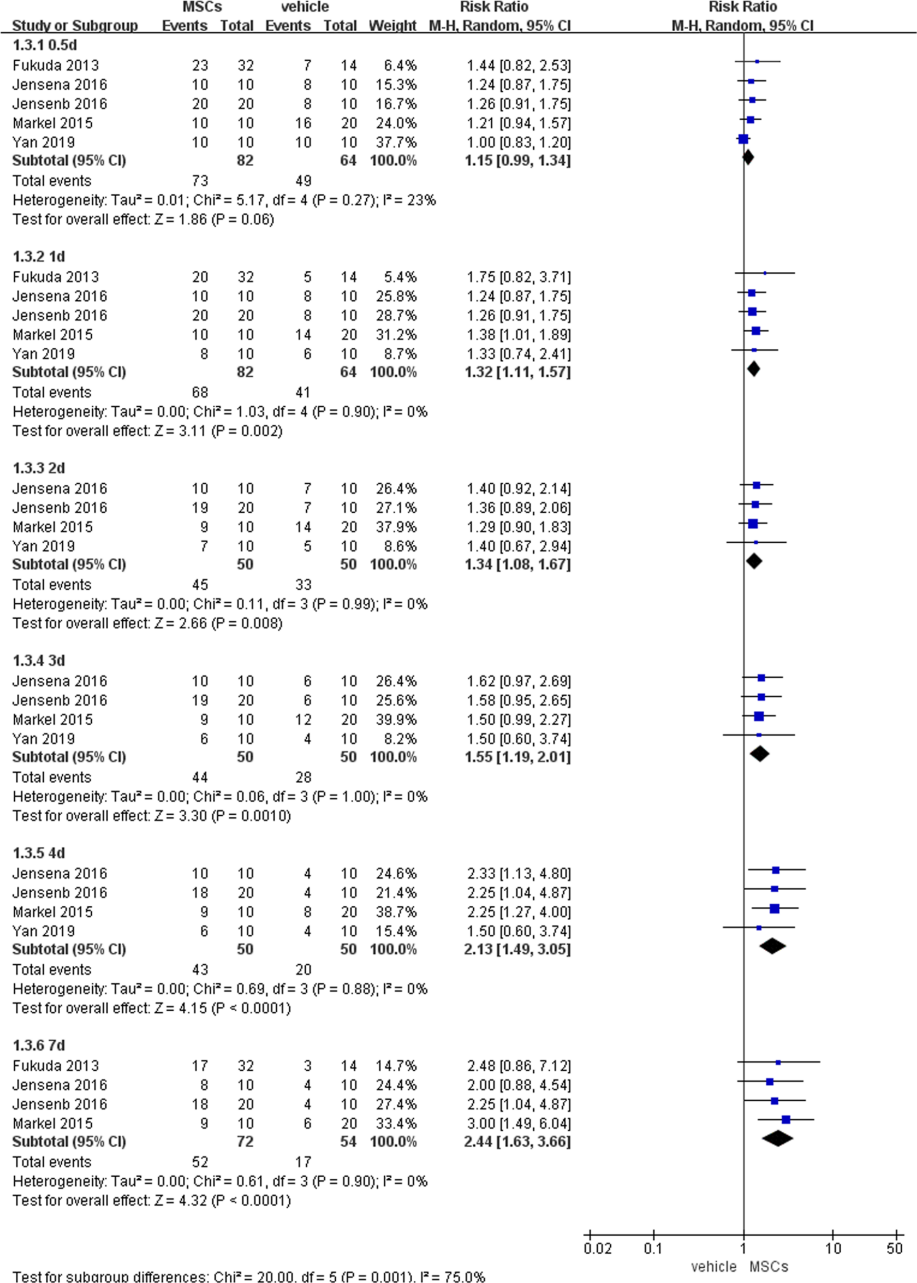
Primary outcome of survival rate at 6 different time points

The first goal of this study was to evaluate whether MSCs can improve the survival of animals with intestinal IRI. We divided the results into 6 subgroups according to reperfusion for different time (0.5, 1, 2, 3, 4 and 7 d following reperfusion). Increased survival was noted in the MSCs-treated group compared to the vehicle-treated group (1.32, 95% CI 1.11–1.57, *P* = 0.002) at 1 d after reperfusion. At 2 d (1.34, 95% CI 1.08–1.67, *P* = 0.008), 3 d (1.55, 95% CI 1.19–2.01, *P* = 0.001), 4 d (2.13, 95% CI 1.49–3.05, *P* < 0.0001), and 7 d (2.44, 95% CI 1.63–3.66, *P* < 0.0001) after reperfusion, the outcome shared similar significance with the outcome at 1 d after reperfusion. at (Fig. 4).

#### Local intestinal injury

To determine whether MSCs have the therapeutic potential for intestinal IRI, we further characterized the anti-inflammatory and anti-oxidative effects of MSCs by analyzing the levels of interleukin (IL)-6, tumor necrosis factor (TNF)-α, and malondialdehyde (MDA) in the gut.

##### Chiu’s score

**Fig. 5:**
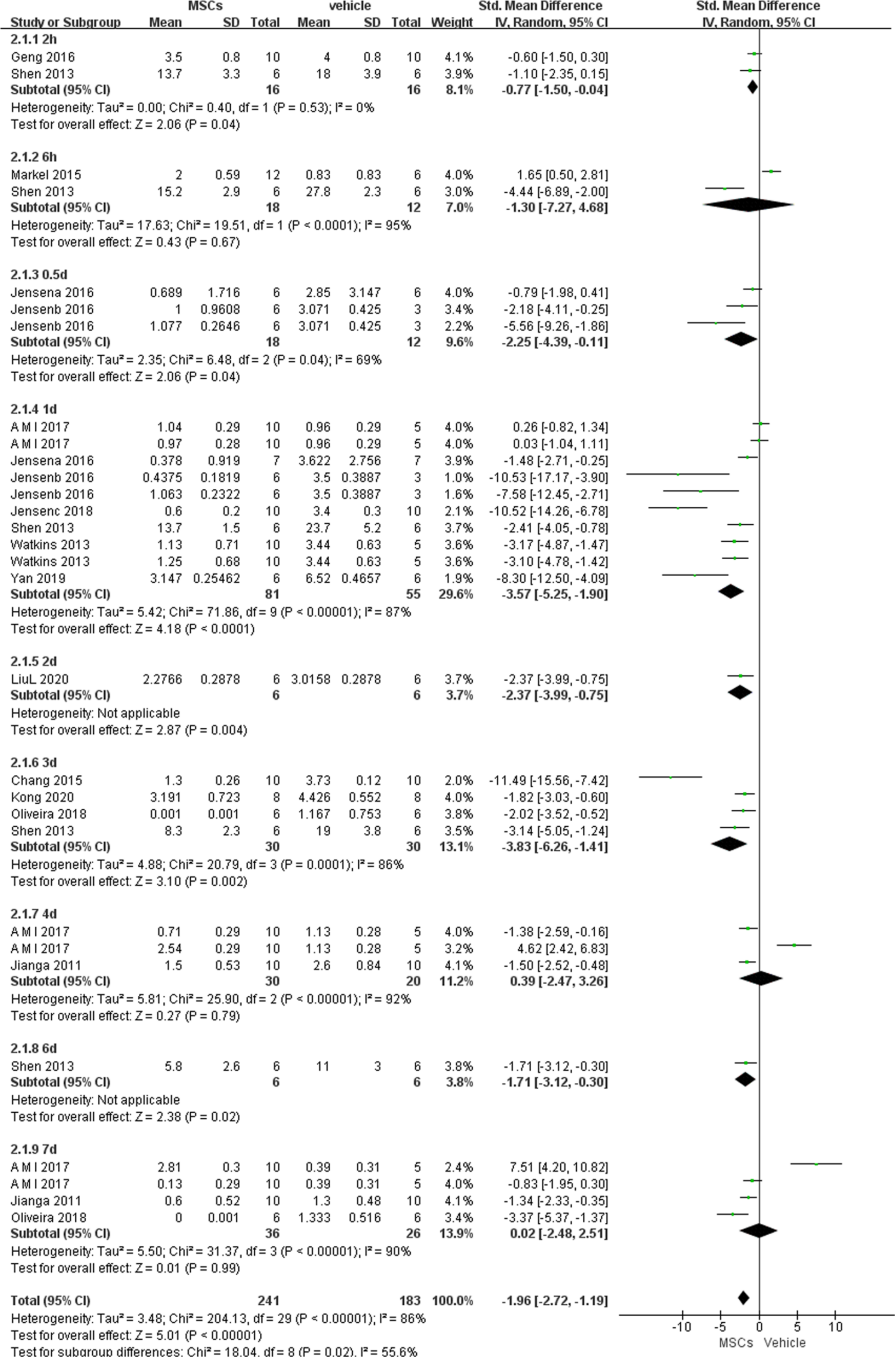
Primary outcome of Chiu’s score at 9 different time points

The severity of intestinal mucosa damage after intestinal IRI was graded using Chiu's score [35]. We analyzed the results reported by 13 studies at different time-points after reperfusion, including 2 h, 6 h, 0.5 d, 1 d, 2 d, 3 d, 4 d, 6 d and 7 d after reperfusion. Similarly, we found histological grades of intestinal injury were negatively associated with the use of MSCs at 2 h (− 0.77, 95% CI − 1.5 to − 0.04, *P* = 0.04), 0.5 d (− 2.25, 95% CI − 4.39 to − 0.11, *P* = 0.04), 1 d (− 3.57, 95% CI − 5.25 to − 1.9, *P* < 0.0001), and 3 d (− 3.83, 95% CI − 6.26 to − 1.41, *P* = 0.002) after reperfusion (Fig. 5).

##### Intestinal IL-6 and TNF-α levels

**Fig. 6:**
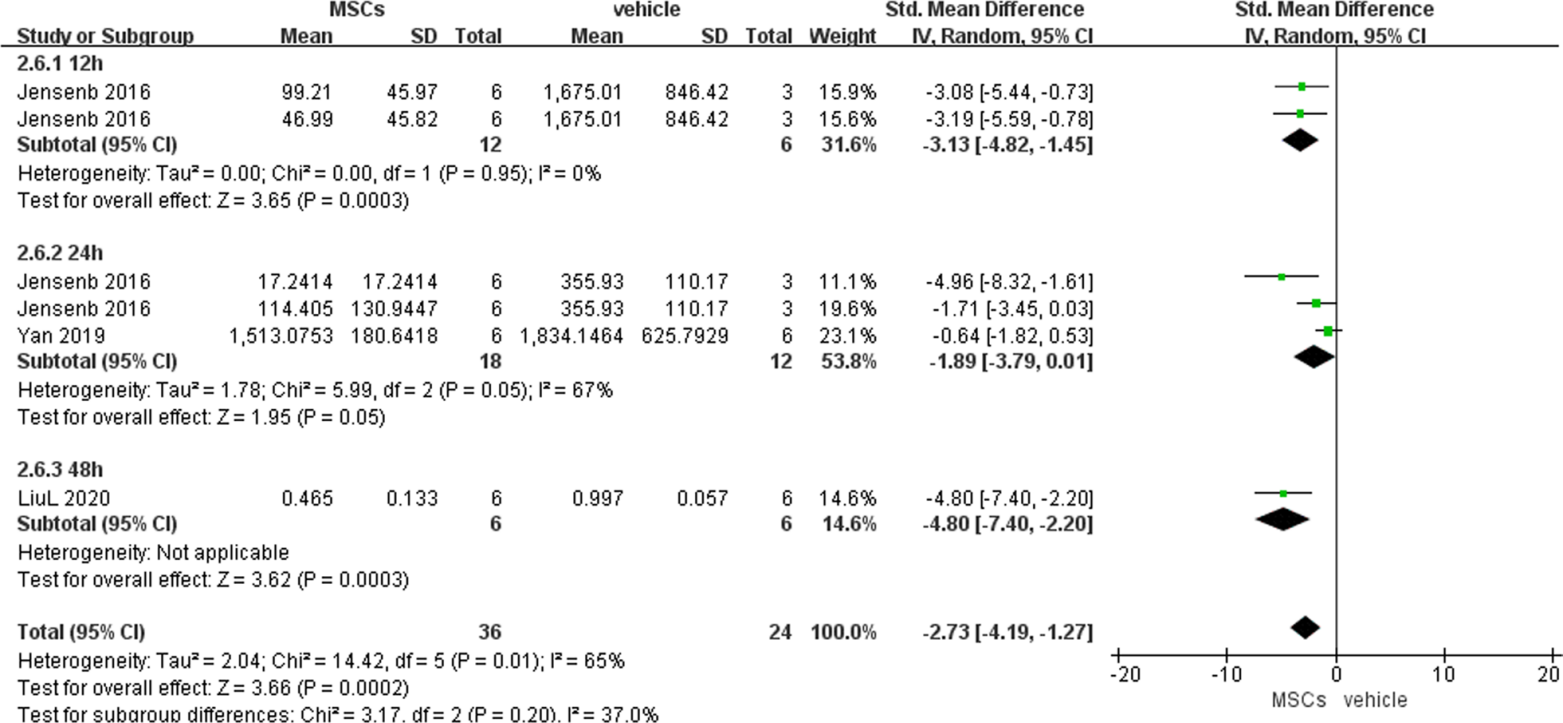
Primary outcome of intestinal IL-6 level at 3 different time points

**Fig. 7:**
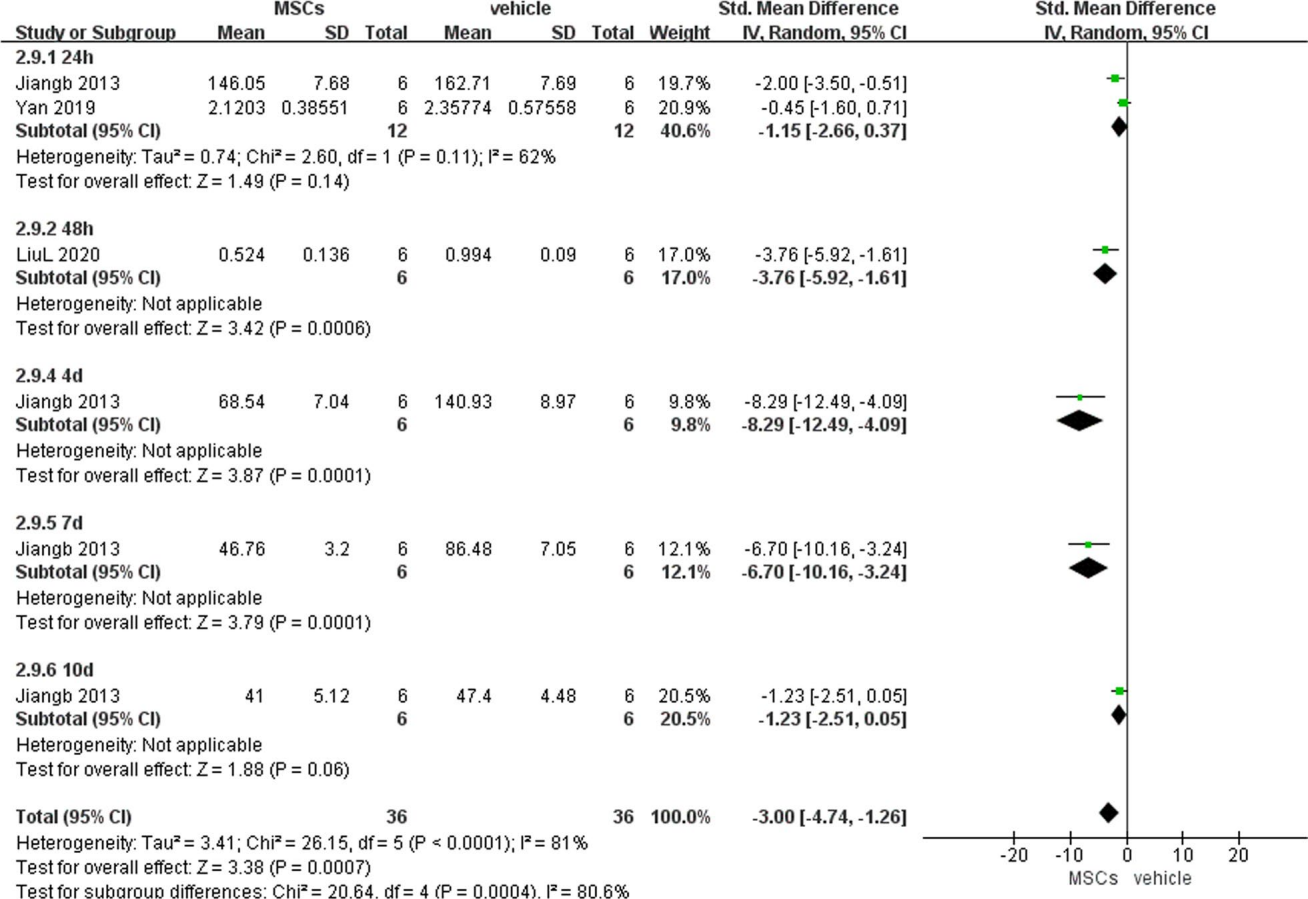
Primary outcome of intestinal TNF-α level at 5 different time points

We pooled the level of intestinal IL-6 or TNF-α at different time points, since they were only reported by 3 articles. Significantly decreased intestinal IL-6 (− 2.73, 95% CI − 4.19 to − 1.27, *P* = 0.0002) and TNF-α (− 3.00, 95% CI − 4.74 to − 1.26, *P* = 0.0007) were noted in the experimental group compared with the vehicle group (Figs. 6 and 7).

##### Intestinal MDA level

**Fig. 8:**
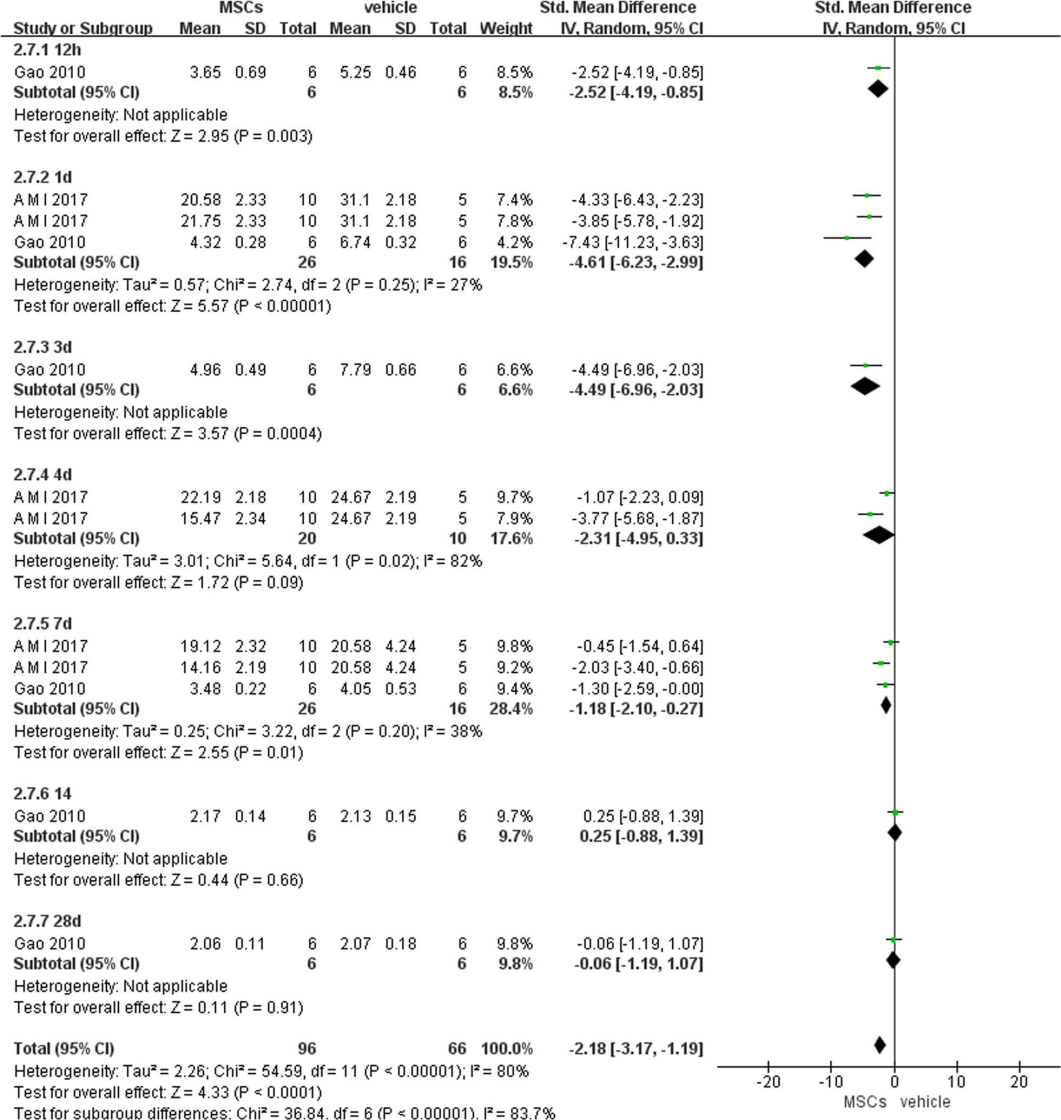
Primary outcome of intestinal MDA level at 7 different time points

Similarly, all data about intestinal MDA were analyzed together, which indicated that MSCs exerted a higher anti-oxidative effect than vehicle (− 2.18, 95% CI − 3.17 to − 1.19, *P* < 0.0001) (Fig. 8).

#### Intestinal barrier dysfunction

The intestinal barrier function or intestinal permeability was evaluated by serum diamine oxidase (DAO), D-Lactate, and TNF-α levels.

##### Serum DAO level

**Fig. 9:**
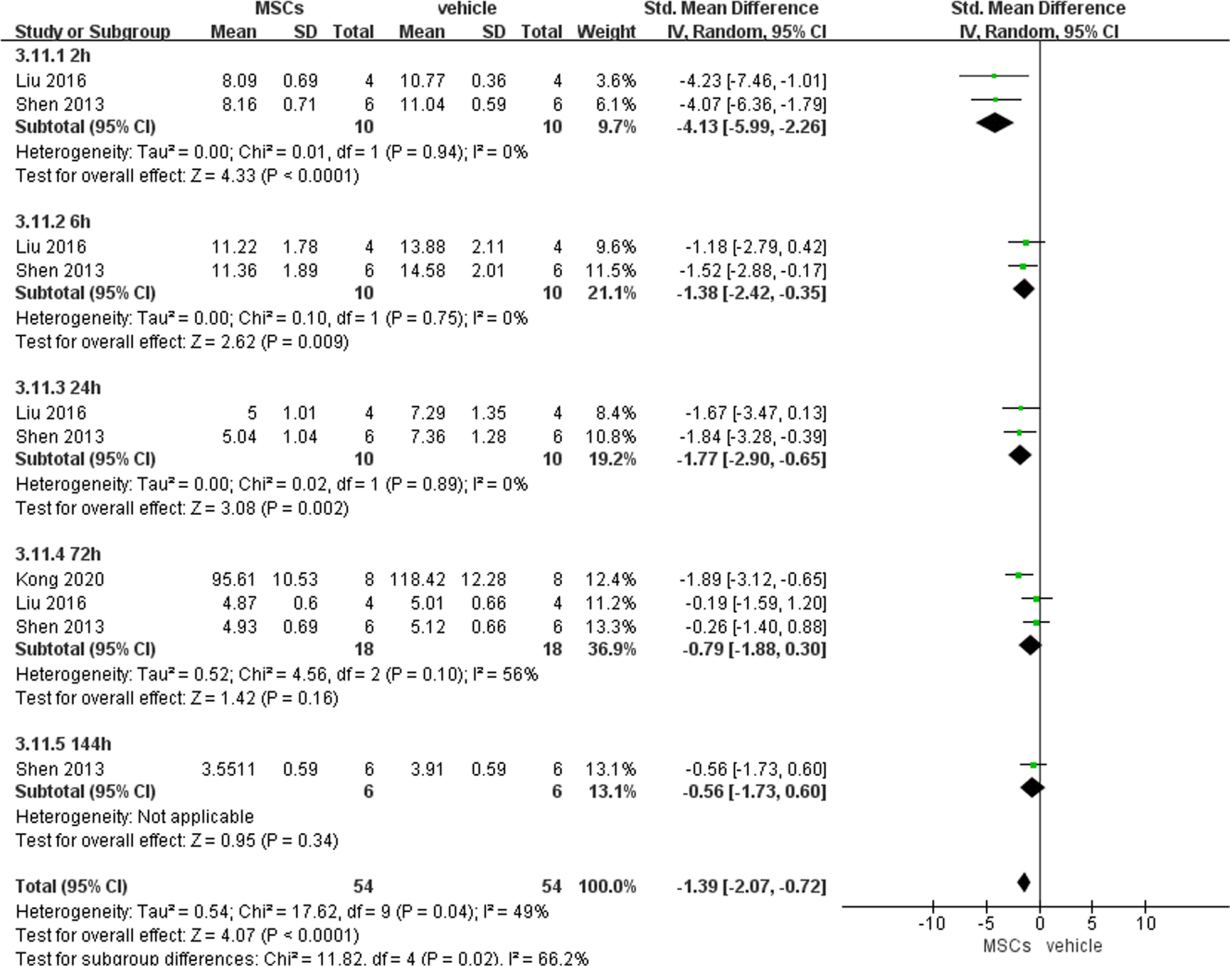
Primary outcome of serum DAO level at 5 different time points

Three studies, including 108 animals with intestinal IRI, reported serum DAO level. We discovered serum DAO level was lower after MSCs administration than vehicle administration at 2 h (− 4.13, 95% CI − 5.99 to − 2.26, *P* < 0.0001), 6 h (− 1.38, 95% CI − 2.42 to − 0.35, *P* = 0.009), and 24 h (− 1.77, 95% CI − 2.9 to − 0.65, *P* = 0.002) after reperfusion (Fig. 9).

##### Serum D-Lactate level

**Fig. 10:**
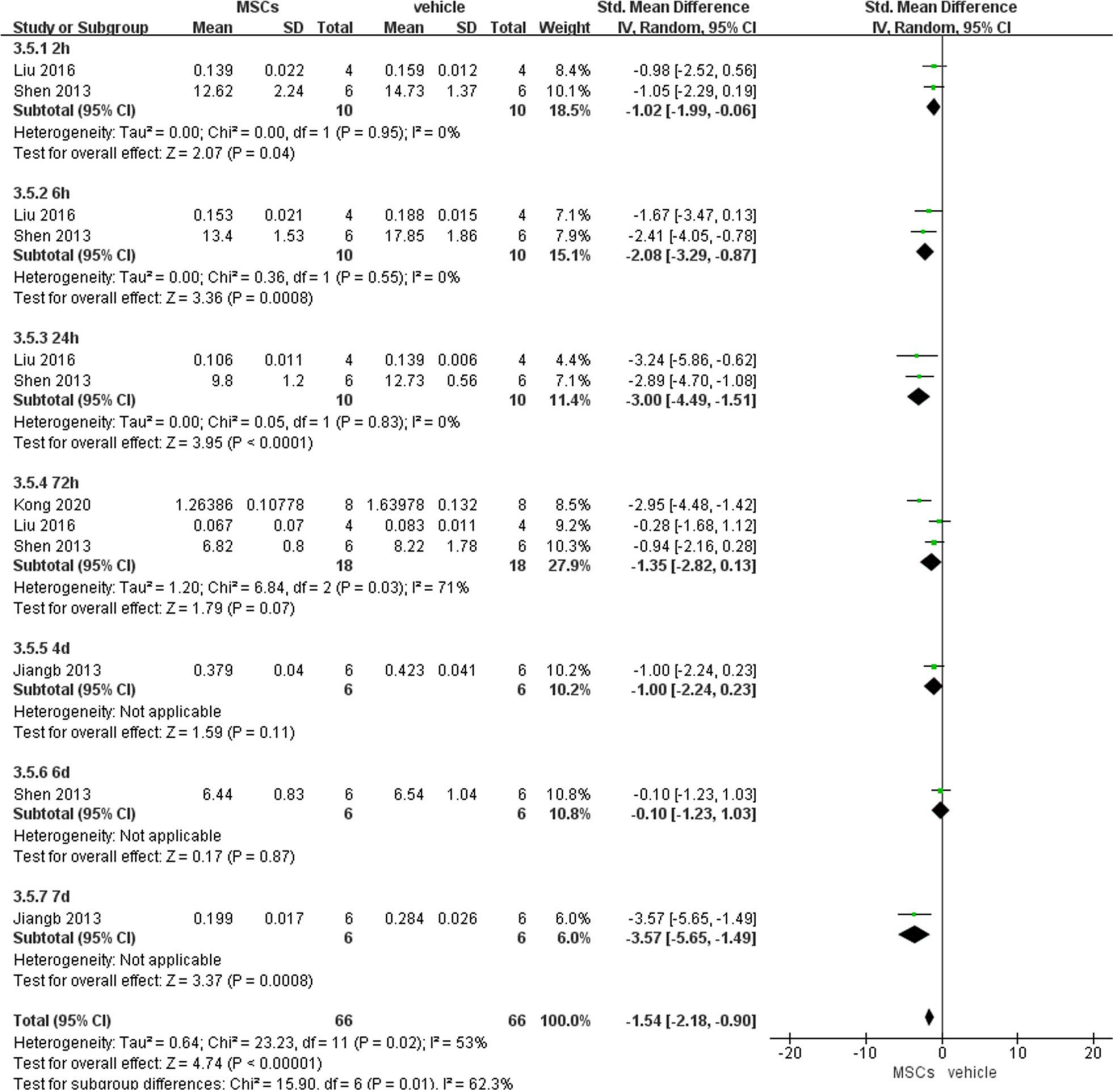
Primary outcome of serum D-Lactate level at 7 different time points

MSCs showed a better effect on reduction in serum D-Lactate than vehicle at 2 h (− 1.02, 95% CI − 1.99 to − 0.06, *P* = 0.04), 6 h (− 2.08, 95% CI − 3.29 to − 0.87, *P* = 0.0008), and 24 h (− 3.00, 95% CI − 4.49 to − 1.51, *P* < 0.0001) after reperfusion (Fig. 10).

##### Serum TNF-α level

**Fig. 11:**
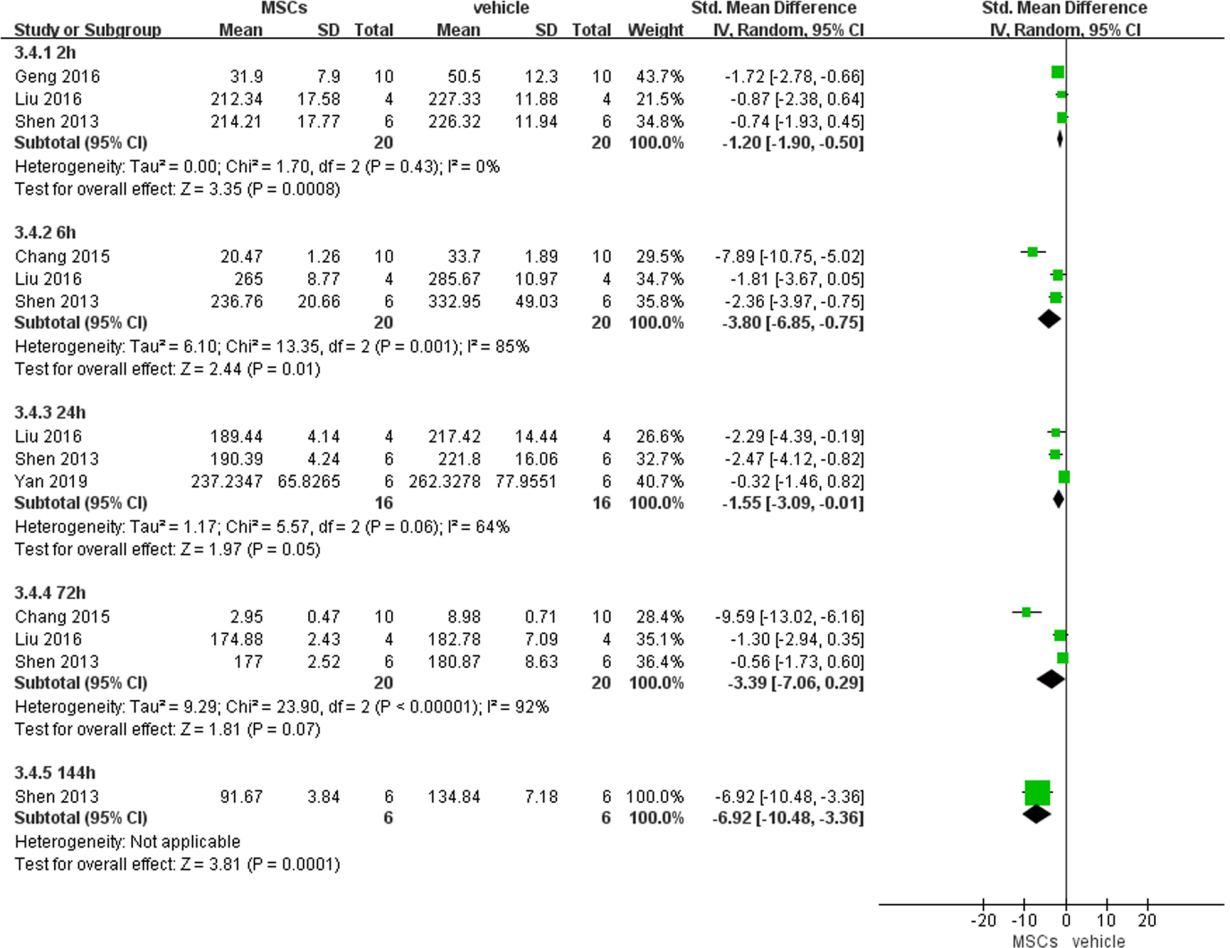
Primary outcome of serum TNF-α level at 5 different time points

We observed a significant difference in serum TNF-α level between MSCs and vehicle only at 2 h (− 1.20, 95% CI − 1.90 to − 0.5, *P* = 0.0008) and 6 h (− 3.80, 95% CI − 6.85 to − 0.75, *P* = 0.01) after reperfusion. Although non-significance was discovered at 1 d, 3 d after reperfusion, we noted the *P*-value (*P* = 0.05, 0.07, respectively) approached statistical significance (*P* < 0.05), which suggested MSCs had an inhibitory effect on serum TNF-α (Fig. 11).

## Discussion

### Summary of evidence

As far as we know, it was the first study to pool all available evidence and show the beneficial effect of MSCs against intestinal IRI. Eighteen studies compared MSCs to placebo controls were enrolled. Although meta-analyses of animal studies were not common, they were recommended when intended to provide general guidance for clinical endeavors. Our meta-analysis showed MSCs therapy was correlated with alleviated pathology injury (decreasing Chiu’s score), reduced inflammation (decreasing IL-6 and TNF-α) and oxidative stress (decreasing MDA), and improved intestinal barrier function (decreasing serum DAO, D-Lactate and TNF-α) in the setting of IRI-induced intestinal damage.

### The possible mechanism for the effect of MSCs in intestinal IRI

Despite intensive investigation on intestinal IRI, its pathogenesis so far has not been entirely clarified. The pathogenesis is believed to be multifactorial, including promoting leukocyte adhesion, generating reactive oxygen species (ROS), releasing mediators of immunological dysregulation, endothelial cell damage, energy exhaustion, and intracellular calcium overload [15, 36]. Although IECs can survive temporary periods of hypoxia, severe hypoxia or continuously block of the blood supply can cause irreversible damage to the intestine tissue. Following severe IRI, intestinal barrier dysfunction occurs due to IECs loss and intercellular tight junctions (TJ) disruption. Gut barrier breakdown contributes to toxic macromolecules, bacteria, and cytokines from the gut lumen into the systemic circulation. While in this situation, MSCs can function as a protective molecule.

Though it is clear that MSCs have protective effects against intestinal IRI, their mechanism of action of MSCs is not completely clear. These mechanisms mainly fall into two categories: (1) exogenous MSCs migrate into the damaged intestinal tissue and differentiate into IECs to enhance the integrity of gut barrier; (2) exogenous MSCs protect IECs through the release of paracrine and/or endocrine cytokines with pleiotropic effects, including anti-inflammation, anti-oxidation, anti-apoptosis, promotion of cell proliferation, and pro-angiogenesis. However, it has been well documented that the former has little efficacy, and only a few MSCs reach target tissues after intravenous injection [37, 38]. Hence, the latter deserves further elaboration. (Table 2).

**Table 2:** The proposed molecular mechanism of the protective effect of MSCs for intestinal IRI

References	Mechanism	Effect
AMI et al. [18]	Oxidative stress, inflammation, and proliferation	Decreased intestinal MDA, TNF-α, IL-6, IL-1β, TGF-β1, MPO, MIP-2; increased intestinal EP3, IL-1Ra, PCNA
Chang et al. [4]	Oxidative stress, inflammation, apoptosis, and proliferation	Decreased intestinal NOX-1, NOX-2, TNF-α, MPO, NF-κB, MMP-9, iNOS, Bax, caspase-3, PCNA; increased intestinal NQO-1, GR, GPx, HO-1
Fukuda et al. [19]	Inflammation; intestinal barrier function	Decreased plasma IL-6; increased plasma IL-10
Gao et al. [20]	Oxidative stress	Decreased intestinal MDA; increased intestinal SOD
Geng et al. [21]	Inflammation, intestinal barrier function, and proliferation	Decreased intestinal NF-κB, serum TNF-α, IL-6; increased intestinal SDF-1, CXCR-4, EGF, EGFR
Jensena et al. [22]	Inflammation and tight junction	Decreased intestinal GCSF; increased claudin-1
Jensenb et al. [23]	Inflammation	Decreased intestinal IL-6, MIP-1α, MIP-2α, and IP-10
Jensen et al. [24]	Unclear	Improved histologic mucosal injury
Jiang et al. [25]	Intestinal barrier function	Decreased serum D-Lactate, urine Lactulose/Mannitol ratio, and incidence of enteric bacterial translocation
Jiang et al. [26]	Inflammation and proliferation	Decreased intestinal TNF-α, NF-κB; increased intestinal PCNA; induced phosphorylation of ERK1/2
Kong et al. [27]	Inflammation, intestinal barrier function, and pyroptosis	Seemed to decrease serum DAO, D-Lactate, IL-1β, intestinal IL-1β, TNF-α, IL-6; seemed to inhibit pyroptosis (NLRP3/caspase-1/IL-18)
Liu and Li [28]	Inflammation, intestinal barrier function and tight junction	Decreased serum DAO, D-Lactate, TNF-α; increased ZO-1
Liu et al. [29]	Inflammation and apoptosis	Decreased intestinal MPO, TNF-α, IL-6; inhibited phosphorylation of NF-κB-p65, ERK, AKT; activated COX-2-PGE_2_ signaling
Markel et al. [30]	Inflammation	Decreased intestinal sALK-1, betacellulin, endothelin; increased intestinal Eotaxin, MIG, MCP-1, IP-10
Oliveira et al. [31]	Inflammation	Decreased intestinal polymorphonuclear inflammatory cells; improved histologic mucosal injury
Shen et al. [32]	Intestinal barrier function and tight junction	Decreased serum DAO, D-Lactate, TNF-α; increased ZO-1
Watkins et al. [33]	Unclear	Improved histologic mucosal injury
Yan et al. [34]	Inflammation and intestinal barrier function	Decreased serum IL-6

IL-1β, -6, -10, 18: interleukin-1β, -6, -10, -18; TGF-β1: transforming growth factor-β1; TNF-α, tumor necrosis factor-α; IL-1Ra: interleukin-1 receptor antagonist; IP-10: interferon-γ-inducible protein-10; iNOS: inducible nitric oxide synthase; SDF-1: stromal-derived factor-1; EGF: epidermal growth factor; MCP-1: monocyte chemoattractant protein-1; NOX: nicotinamide adenine dinucleotide phosphatase oxidase; HO: heme oxygenase; caspase-3: cysteinyl aspartate-specific proteinase; ERK 1/2: extracellular regulated protein kinases; NF-κB: nuclear factor; DAO: diamine oxidase; MDA: malondialdehyde; ZO-1: zonula occluden-1; NLRP3: NOD-like receptor protein 3; Bax: B-cell lymphoma-2-associated X protein

#### Protective effect of MSCs related to inflammation

When intestine tissues are damaged by IRI, leukocyte infiltration mediated by leukocyte adhesion to endothelial cells results in microcirculatory disturbances, leading to a further cascade of post-ischemic intestinal inflammation and exacerbating the tissue injury [39]. Intestinal TNF-α and IL-6 are used as markers of intestinal local inflammation and evaluated in this research because of their involvement in pathological hyperinflammatory states. MSCs release many types of cytokines factors through paracrine effects or directly interacts with immune cells, leading to immunomodulation. The included studies showed that MSCs positively contributed to recovery process by decreasing pro-inflammatory cytokines (TNF-α, IL-6, IL-1β, TGF-β1, MPO, NF-κB, and iNOS) [4, 18, 21, 23, 26, 27, 29] and increasing anti-inflammatory cytokines (EP3 and IL-1Ra) [18] following IRI in the intestine of animals.

The Toll/Interleukin-1 receptor (TIR) domain is highly conserved among all toll-like receptors (TLRs) and triggers the TLR-mediated signaling pathways. TLRs are recognized and combined with corresponding ligands, such as myeloid differentiation primary response gene 88 (MyD88) and TIR-domain-containing adaptor-inducing interferon-β (TRIF), and consequently activate downstream signaling pathways, including the transcription factor nuclear factor (NF)-κB and the mitogen-activated protein (MAP) kinase (MAPK) pathways [40, 41]. Both of the two are crucial inflammation-associated pathways, and they play pivotal roles in intestinal inflammatory response [42]. NF-κB pathway regulates immune and inflammatory responses. In response to specific signals, the NF-κB dimer translocates from its resting state in the cytoplasm to the nucleus, where it regulates the transcription of target genes, such as TNF-α, IL-1β, and IL-6 [43]. MSCs were found to have the anti-inflammatory effect by inhibiting NF-κB signaling pathway [4, 21, 26, 29].

MAPK pathway consists of three components: MAP kinase kinase kinase (MAP3K), MAP kinase kinase (MAP2K), and MAPK (ERK1/2, p-38MAPK, and JNK). MAPK pathway, activated by external signals, regulates multiple cellular pathways, such as cell proliferation, apoptosis, inflammation, and cytokine/chemokine production [44]. Surprisingly, Jiang et al. [26] and Liu et al. [29] found MSCs for intestinal IRI had opposite effects on ERK1/2 pathway. The possible reason for this was that they used different animals and MSCs treatment (Table 2). Hence, the mechanism of this effect requires further study.

#### Protective effect of MSCs related to oxidation

Oxidative stress is characterized by a severe imbalance of oxidative and antioxidant systems [45], which plays a principal role in the pathogenesis of IRI, especially in the reperfusion phase. And its relationship with intestinal IRI has been widely recognized and extensively studied. We analyzed cellar oxidant activity during intestinal injury using measuring intestinal MDA, one of highly reactive dicarbonyls generated by lipid peroxidation [46]. Our results [4, 18, 20] showed MSCs could significantly treat local oxidation in IRI-induced intestinal mucosa (decreasing MDA, NOX-1, and NOX-2; increasing SOD, NQO-1, GR, GPx, and HO-1).

Nicotinamide adenine dinucleotide phosphatase (NADPH) oxidases (NOX) family (mainly including NOX1, 2, and 4) mediates the production of ROS to participate in intestinal mucosal barrier damage [47]. Heme oxygenase (HO), an essential stress response gene, to date, has three isoforms: HO-1 (inducible), HO-2 (constitutive) and HO-3 (constitutive). Additionally, HO-1 is of particular interest because it plays a central role in cellular antioxidant defenses. MSCs could exert antioxidant effects by down-regulation of NOX pathway and up-regulation of HO-1 pathway [4].

#### Protective effect of MSCs related to programmed IECs death

Intestinal IRI can trigger different types of IECs death, which are categorized into non-programmed and programmed cell death. The former refers to necrosis (a passive, accidental, and unregulated cell death), and the latter generally consists of apoptosis, necroptosis (or programmed necrosis), pyroptosis, ferroptosis, and autophagy [48, 49].

B-cell lymphoma-2 (Bcl-2)-associated X protein (Bax), existing in the cytosol, is an inactive, globular protein and directly activated by pro-apoptotic stimuli, contributing to cell death. Its specific biological process is that activated Bax/Bak forms pores in the outer mitochondrial membrane, releasing apoptogenic factors (including cytochrome c) from mitochondria into the cytosol to activate the cysteinyl aspartate-specific proteinase (caspase) cascade, eventually inducing cell death. A higher level of Bax/Bcl-2 (an apoptosis-inhibiting protein) ratio suggests a strong pro-apoptotic activity. Chang et al. [4] found MSCs inhibited apoptosis in IRI-induced IECs, potentially through inhibition of Bax and cleaved caspase-3.

Being distinct from apoptosis, pyroptosis is a newly discovered programmed cell death process resulting from inflammatory assault, and it occurs in multiple tissues [50, 51]. It is characterized by cellular swelling, plasma membrane rupture, release of pro-inflammatory intracellular contents (such as IL-1β, IL-18) into the extracellular milieu [52, 53]. The main process of pyroptosis includes the formation of the NOD-like receptor protein 3 (NLRP3) inflammasome, which consists of the sensor molecule NLRP3, the adapter protein apoptosis-associated speck-like protein containing a caspase recruitment domain (ASC), and pro-caspase-1 [54]. Thus, Kong et al. [27] concluded that MSCs protected from pyroptosis in IRI of the intestinal, possibly via the NLRP3/caspase-1/IL-18 pathway.

#### Protective effect of MSCs related to intestinal barrier

##### Intestinal barrier structure (TJ)

Intestinal IRI causes not only local injury but multiple organs failure by the impaired intestinal barrier. Intestinal barrier dysfunction is widely regarded as a major cause of many complications of intestinal IRI. The intestinal barrier, consisting of mechanical, chemical, and biological barriers, protects tissues from the invasion of external harmful substances in living organisms. The formation and maintenance of TJ between IECs is crucial to maintain barrier function and regulate intestinal permeability [55]. MSCs preserved intestinal barrier function by decreasing TJ permeability, including TJ transmembrane protein, claudin-1 [22], and TJ scaffolding protein, zonula occluden (ZO)-1 [28, 32].

##### Intestinal barrier function

Impaired barrier function results in the movement of the luminal toxins and antigens material into the circulation, causing SIRS. The intestinal barrier dysfunction assay was conducted using serum DAO, D-lactate, and TNF-α. DAO, existing in IECs of mammalian, is a highly active intracellular enzyme [56]. Also, D-lactate is an end product of metabolism of intestinal bacteria in the gastrointestinal tract, and mammals can neither produce nor catabolize it. Only when intestinal permeability is greatly increased, D-lactate can enter circulating blood [57]. Subsequently, MSCs preserved intestinal barrier function [21, 25, 27, 28, 32, 34].

### Advantage and limitation of this review

The advantages of this review are apparent. First, we are the first to conduct a meta-analysis of the beneficial effects of MSCs therapy on intestinal IRI preclinical models. Second, we conducted a systematic literature search and summarized the potential mechanisms of MSCs against intestinal IRI, contributing to provide a new effective approach for clinical prevention and treatment of intestinal IRI.

Inevitably, the article also has some limitations. First, although the included studies aimed to explore the relationship between MSCs and intestinal IRI, they used different animals (species, gender, age, weight), disease models (duration of SMA occlusion and deocclusion), and MSCs treatment (type, dosage, administration route and timing), which resulted in inevitably significant high heterogeneity across the pooled results. Second, we estimated pooled relative risks using random-effects models. Third, when data were only presented graphically, we digitized the data using GetData Graph Digitizer 2.24. Finally, sensitivity analysis was not performed when there was significant heterogeneity.

## Conclusion and future perspectives

**Fig. 12:**
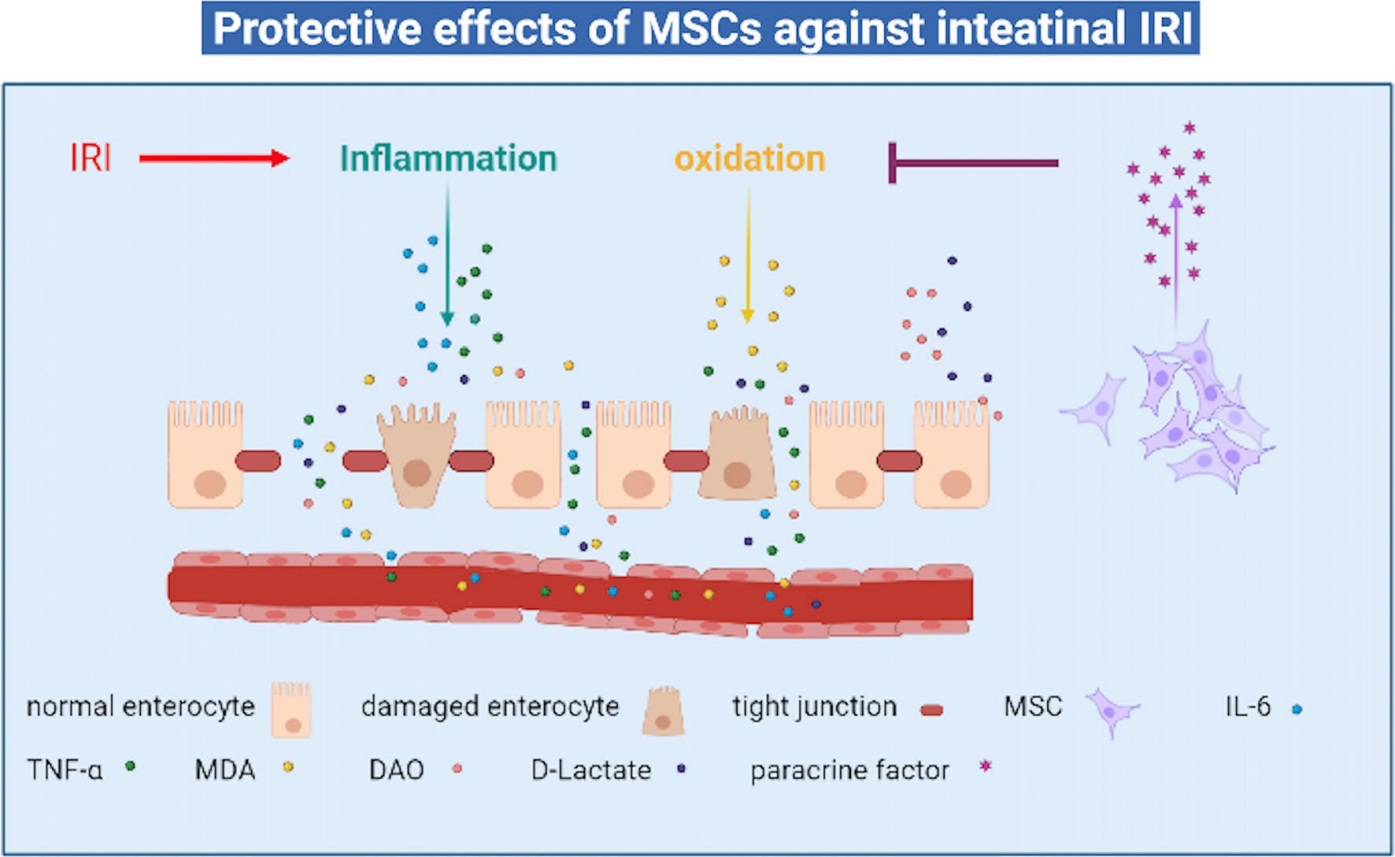
The protective effect of MSCs for intestinal IRI

In summary, this systemic review and meta-analysis firstly evaluate the effects of MSCs against intestinal IRI in animal models. The outcome suggests that MSCs therapy leads to attenuating intestinal injury and promoting intestinal barrier function, providing important clues for future research and clinical trials. The possible mechanism is that it can inhibit inflammation, oxidation, apoptosis, pyroptosis and finally preserve intestinal barrier function. MSCs could be a promising therapy to treat intestinal IRI (Fig. 12).

Nonetheless, studies on the specific targets and regulatory mechanisms are still in the preliminary stage, and the precise mechanism of protection is not fully understood. Additionally, only few studies have evaluated the relationship between MSCs and different types of regulated cell death, such as necroptosis, ferroptosis, and autophagy, which are closely correlated with intestinal IRI. Moreover, research on the protective effects of MSCs in intestinal IRI is limited to basic experiments such as those on cells and animals, and there is no correlated clinical research about them. Therefore, more in-depth studies on MSCs should be conducted to explore the mechanism of this effect. Also, with the deepening of research, exosomes isolated from MSCs (MSCs-Exo) have been of great interest to the scientific community. MSCs-Exo exert the similar biologic effects with MSCs, and their major advantage is their non-immunogenic nature, leading to a long and stable circulation. Exosomes contain non-coding RNAs (ncRNAs), such as microRNAs (miRNAs) and long non-coding RNAs (lncRNAs), which can be sequenced and profiled in order for diagnosis of disease. The role of MSCs-Exo and the regulatory function of MSCs-derived exosomal ncRNAs in intestinal IRI still need to be further addressed.

## Supplementary Information

**Additional file 1. **Search strategy.

## Data Availability

Not applicable.

## Abbreviations

Caspase: Cysteinyl aspartate-specific proteinase
DAO: Diamine oxidase
HO: Heme oxygenase
IECs: Intestinal epithelial cells
IL-6: Interleukin-6
IRI: Ischemia–reperfusion injury
MDA: Malondialdehyde
MSCs: Mesenchymal stem cells
MSCs-Exo: MSCs-derived exosomes
NF-κB: Nuclear factor-κB
NOX: Nicotinamide adenine dinucleotide phosphatase oxidase
ERK1/2: Extracellular regulated protein kinases ½
SIRS: Systemic inflammatory response syndrome
SMA: Superior mesenteric artery
TNF-α: Tumor necrosis factor-α
ZO-1: Zonula occluden-1
