# Supplementary material for: Mesenchymal stem cells against intestinal ischemia–reperfusion injury: a systematic review and meta-analysis of preclinical studies
Source: Stem Cell Res Ther. 2022 May 26;13:216. doi: 10.1186/s13287-022-02896-y (PMC9137086; doi:10.1186/s13287-022-02896-y)
Supplement: Supplementary file 1 — Additional file 1. Search strategy. [file 13287_2022_2896_MOESM1_ESM.docx]

**Search strategy:**

**Pubmed: 45 records**

#1: ("Intestines"[Mesh]) OR (intestin*[Title/Abstract])

#2: ((((Mesenchymal Stem Cell*[Title/Abstract]) OR (Mesenchymal Stromal Cell*[Title/Abstract]) OR (Mesenchymal Progenitor Cell*[Title/Abstract]) OR (Bone Marrow Stromal*[Title/Abstract]) OR ((Wharton*[Title/Abstract]) AND (Jelly Cell*[Title/Abstract]))) OR ("Mesenchymal Stem Cells"[Mesh])

#3: ((("Ischemia"[Mesh]) OR ((ischaemi*[Title/Abstract]) OR (ischemi*[Title/Abstract]) OR (reperfusion[Title/Abstract])))

#4: (Reperfusion*[Title/Abstract]) OR ("Reperfusion"[Mesh])

#5: #3 OR #4

#6: #1 AND #2 AND #5

**Emabse: 57**

#1: (‘intestine’)/exp OR (intestine$):ti,ab,tw

#2: (‘ischemia’/exp OR (ischemi$):ti,ab,kw OR (ischaemi$):ti,ab,kw OR (reperfusion):ti,ab,kw

#3: (‘reperfusion’)/exp OR (reperfusion$): ab,ti

#4: (‘mesenchymal stem cell’)/exp OR (‘mesenchymal stem cell$’):ti,ab,kw OR (‘mesenchymal stromal cell$’):ti,ab,kw OR (‘mesenchymal progenitor cell$’):ti,ab,kw OR ((wharton$):ti,ab,kw AND ‘jelly cell$’):ti,ab,kw) OR (‘bone marrow stromal$’):ti,ab,kw

#5: #2 OR #3

#6: #1 AND #5 AND #4 AND ‘nonhuman’/de

**Cochrane Library: 0 records**

**Web of Science: 74 records**

#1: TS=(“Mesenchymal Stem Cell*” OR “Mesenchymal Stromal Cell*” OR “Mesenchymal Progenitor Cell*” OR “Bone Marrow Stromal*” OR “Wharton* AND “Jelly Cell*””)

#2: TS=(“Intestinal ischemia reperfusion”)

#3: #1 AND #2
